# Diagnostic Performance of PSMA PET/MRI in Characterizing LI-RADS 3 Observations in Patients with Cirrhosis

**DOI:** 10.2967/jnumed.125.271228

**Published:** 2026-04

**Authors:** Onofrio Antonio Catalano, Irun Bhan, Luigi Asmundo, William Robert Bradley, Mattia Fonderico, M. Lisa Zhang, Amirkasra Mojtahed, Mark A. Anderson, Alexander Herold, Azadeh Hajati, Valeria Pena-Trujillo, Peter D. Caravan, Samantha G. Harrington, Shadi Abdar Esfahani, Umar Mahmood, Nahel Elias, Elizabeth P. Walsh, Daniel S. Pratt, Abigail B. Scherrer, Haley Ellis, Emily D. Bethea, Claude Sirlin, Avinash Ramesh Kambadakone, David Ryan, Kelsey Lau-Min

**Affiliations:** 1 Department of Radiology, Massachusetts General Hospital, Harvard Medical School, Boston, Massachusetts;; 2 Department of Gastroenterology, Massachusetts General Hospital, Harvard Medical School, Boston, Massachusetts;; 3 Department of Radiology, Ospedale Niguarda Ca’ Granda, Milano, Italy;; 4 Department of Neurology, Biostatistics, Azienda USL Toscana Nordovest, Ospedale ‘Villamarina’, Piombino, Italy;; 5 Department of Pathology, Massachusetts General Hospital, Harvard Medical School, Boston, Massachusetts;; 6 Department of Transplant Surgery, Massachusetts General Hospital, Harvard Medical School, Boston, Massachusetts;; 7 Department of Oncology, Massachusetts General Hospital, Harvard Medical School, Boston, Massachusetts; and; 8 Department of Radiology, Liver Imaging Group, University of California at San Diego, San Diego, California

**Keywords:** [^68^Ga]Ga-PSMA-11 PET MRI, hepatocellular carcinoma, LI-RADS 3, liver cirrhosis, molecular imaging

## Abstract

Liver Imaging Reporting and Data System (LI-RADS) category 3 (LR-3) observations remain indeterminate and often result in repeated follow-up or biopsy. Prostate-specific membrane antigen (PSMA) is overexpressed in hepatocellular carcinoma (HCC) neovasculature and may serve as a useful imaging biomarker. This study aimed to evaluate whether [^68^Ga]Ga-PSMA-11 PET/MRI improved characterization of LR-3 observations in patients with cirrhosis compared with MRI alone. **Methods:** In this prospective study, conducted between March 2022 and June 2024, 19 patients with cirrhosis and 54 LR-3 observations identified on prior MRI underwent [^68^Ga]Ga-PSMA-11 PET/MRI. An observation was classified as HCC if it demonstrated focal ^68^Ga-PSMA uptake greater than background liver combined with at least 1 LI-RADS major or ancillary feature. The reference standard was histopathology or a follow-up MRI within 12 mo. Diagnostic metrics were calculated. Univariable logistic regression and decision tree analysis were performed to identify imaging predictors of malignancy. **Results:** Of the 54 LR-3 observations, 13 (24%) were confirmed as HCC and 41 (76%) as benign. [^68^Ga]Ga-PSMA-11 PET/MRI correctly identified 12 of 13 HCCs (sensitivity, 92%; 95% CI, 66.7–99.6) and 39 of 41 benign observations (specificity, 95%; 95% CI, 81.9–99.3). Overall diagnostic accuracy was 94%, with a positive predictive value of 86% and negative predictive value of 97%. Diagnostic performance was significantly better than MRI alone (McNemar test, *P* < 0.001). [^68^Ga]Ga-PSMA-11 uptake was the only significant imaging predictor of malignancy on univariable analysis (odds ratio, 5.7; *P* = 0.017). Decision tree analysis identified [^68^Ga]Ga-PSMA-11 uptake, observation size, and hepatobiliary phase hypointensity as principal discriminators. **Conclusion:** [^68^Ga]Ga-PSMA-11 PET/MRI demonstrates high diagnostic accuracy in differentiating malignant from benign LR-3 liver observations in patients with cirrhosis. This technique may reduce unnecessary follow-up imaging and biopsy. These results support further validation of [^68^Ga]Ga-PSMA-11 PET/MRI as a promising imaging approach for indeterminate liver observations.

## Visual Abstract

**Figure:**
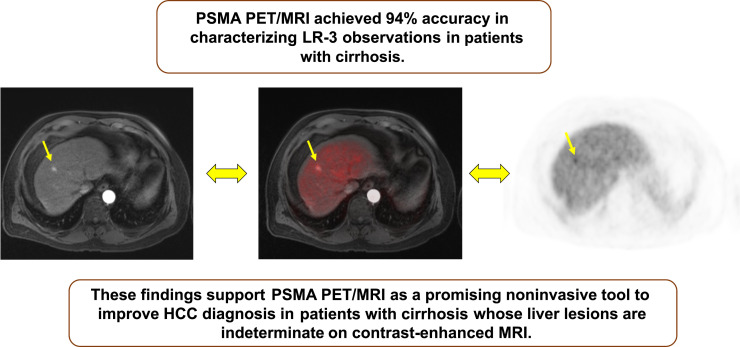

Hepatocellular carcinoma (HCC) is the sixth most commonly diagnosed cancer and the third leading cause of cancer-related death worldwide, with approximately 900,000 new cases annually. Early-stage detection is associated with curative treatment and significantly improved survival, whereas advanced-stage disease has a 5-y survival rate of less than 20% (1). Contrast-enhanced MRI is widely used for HCC surveillance and diagnosis in patients with cirrhosis, offering detailed assessment of vascular and hepatobiliary features (2). However, 20%–40% of examinations reveal indeterminate observations categorized as Liver Imaging Reporting and Data System (LI-RADS) category 3 (LR-3), which carry an intermediate risk of HCC (3–6). Meta-analyses have found that approximately 31% of LR-3 observations progress to HCC, and explant pathology in liver transplant recipients has confirmed HCC in up to 63% of these patients (6,7). Currently, no reliable imaging method exists to determine which LR-3 observations will progress, resulting in repeated imaging, biopsy, financial burden, and patient anxiety (8,9).

Prostate-specific membrane antigen (PSMA), a glutamate carboxypeptidase II, is highly expressed not only in prostate cancer but also in HCC neovasculature. Ex vivo studies have demonstrated PSMA expression in approximately 90% of HCC-associated endothelial cells, with high expression in up to 80% of tumors (10–15). Importantly, PSMA is absent or minimally expressed in benign and dysplastic nodules, liver observations, and hepatic adenomas (16). Clinical studies using [^68^Ga]Ga-PSMA-11 PET/CT have shown promising sensitivity for HCC detection and patient management changes (17–20). Combining PSMA-targeted PET with MRI may offer simultaneous metabolic and morphologic assessment, potentially improving the characterization of indeterminate liver observations.

Given the clinical challenge posed by LR-3 observations and the lack of robust risk stratification tools, a noninvasive imaging method capable of accurately differentiating benign from malignant LR-3 observations would be of substantial clinical value. The aim of this study was to evaluate the diagnostic performance of [^68^Ga]Ga-PSMA-11 PET/MRI in characterizing LR-3 observations in patients with cirrhosis, leveraging the high specificity of neovascular PSMA expression in HCC (16) and the superior diagnostic performance of MRI (21–23).

## MATERIALS AND METHODS

This prospective study is a subanalysis of an institutional review board–approved trial designed to provide a comprehensive single-visit assessment of liver cirrhosis (NCT06265272). The parent study will evaluate hepatic steatosis, iron overload, liver function, hemodynamics, and hepatic observations. Written informed consent was obtained from all enrolled patients as part of the main prospective study, which included this substudy. All procedures complied with the ethical standards of the 2013 Declaration of Helsinki.

### Study Design and Patient Selection

Patients with cirrhosis and LR-3 observations identified on prior contrast-enhanced MRI (using extracellular or hepatobiliary agents) were eligible for inclusion. Enrollment occurred within 3 mo of the index MRI. Cirrhosis was confirmed by clinical, laboratory, and imaging criteria. Inclusion criteria included either histopathologic confirmation of LR-3 observations or a follow-up MRI within 6–12 mo after the PET/MRI study, which were used to determine the reference standard.

Exclusion criteria included systemic therapy or local liver-directed treatments—microwave ablation, radiofrequency ablation, transarterial chemoembolization, selective internal radiation therapy (SIRT), or stereotactic body radiotherapy—within 12 mo before PET/MRI. Patients were also excluded if the index observation had been treated without pathologic proof before imaging follow-up. Local liver-directed therapies performed more than 12 mo before PET/MRI were not an exclusion criterion.

### Imaging Protocol

All studies were performed on a Biograph mMR PET/MRI scanner (Siemens Healthineers). Intravenous [^68^Ga]Ga-PSMA-11 (^68^Ga-gozetotide; Telix Pharmaceuticals) was administered at a dose of 1.8–2.2 MBq/kg. PET acquisition was performed simultaneously with multiparametric MRI over 25 min. The MRI sequences included axial T1-weighted gradient-recalled echo Dixon sequences for attenuation correction; axial and coronal single-shot T2-weighted fast spin-echo sequences; an axial respiratory-gated, fat-saturated T2-weighted fast spin-echo sequence; axial fat-suppressed T1-weighted volumetric interpolated breath-hold examination sequences before and dynamically after contrast injection; and axial diffusion-weighted imaging with *b* values of 50, 400, and 800 s/mm^2^. Gadoxetate disodium (Eovist; Bayer Healthcare) was administered intravenously (0.1 mL/kg body weight, 2 mL/s), followed by a saline flush. Contrast-enhanced images were acquired in the late arterial, portal venous, transitional, and hepatobiliary phases.

### Image Interpretation

Observation interpretation was performed by consensus of 3 radiologists with formal subspecialty training in abdominal radiology and experience with PET/MRI. Readers were provided with prior imaging reports and were asked to locate previously reported LR-3 observations and correlate them with PET/MRI findings.

Two separate analyses were performed at least 4 wk apart and in a random order to reduce recall bias. In the first analysis, [^68^Ga]Ga-PSMA-11 PET/MRI images were evaluated. An observation was classified as HCC only if it demonstrated focal ^68^Ga-PSMA uptake higher than background liver in association with at least 1 LI-RADS major or ancillary feature. Observations without increased ^68^Ga-PSMA uptake were considered benign, regardless of their MRI characteristics. In the second analysis, only the MRI component was assessed independently from PET data. Observations were classified as HCC if they met criteria for LI-RADS category 4 (LR-4) or 5 (LR-5). Observations that remained LR-3 or had completely resolved were considered nonmalignant. For this MRI-only analysis, we evaluated only the MRI part of the PET/MRI.

### Reference Standard

The reference standard was determined by pathology or, for observations without pathology, on follow-up MRI according to LI-RADS version 2018 criteria. In the case of pathology, images were acquired on glass slides at room temperature using an Olympus BX46 microscope (×4 to ×40 objective lenses) and an Olympus DP23 camera. Imaging was performed via Olympus cellSens Entry 3.2 with no fluorochromes or postacquisition processing. Observations were classified as HCC if they developed new major imaging features sufficient to upgrade them to LR-4 or LR-5 or if they demonstrated threshold growth (defined as an increase of ≥ 50% in maximal diameter within ≤ 6 mo). In the absence of these findings, stability or regression over at least 6 mo (up to 12 mo) was considered benign. Both pathology and MRI results were required to be obtained within 12 mo of the [^68^Ga]Ga-PSMA-11 PET/MRI study. If more than 1 follow-up MRI study was obtained within that time frame, the follow-up study that was acquired last or that first demonstrated upgrading or downgrading of a LR-3 observation served as the reference standard. The 12-mo limit was instituted to minimize time-related confounding factors, such as the potential for premalignant observations to develop into HCC (5,24–27).

### Statistical Analysis

Statistical analysis was performed using Python version 3.12.3. Diagnostic performance metrics, including sensitivity, specificity, accuracy, positive predictive value, and negative predictive value, were calculated with 95% CI using the Wilson score interval method. McNemar test was used to compare diagnostic performance between PET/MRI and standalone MRI. The discriminative ability of [^68^Ga]Ga-PSMA-11 PET/MRI was evaluated via receiver-operating-characteristic (ROC) analysis, with area under the curve reported.

We conducted a multivariable generalized linear model including all covariates (radiotracer uptake, observation size, arterial phase enhancement, portal venous washout, enhancing capsule, hepatobiliary phase hypointensity, diffusion restriction) to estimate adjusted effect sizes. To account for clustering of multiple observations within the same patient, we fitted a mixed-effects logistic regression with a random intercept for patient identification. Fixed effects included the same covariates used for the multivariable generalized linear model. Random intercept variance was estimated via Laplace approximation. Wald *z* tests were used for inference on fixed effects (*P* < 0.05). A conditional likelihood ratio test compared the mixed model against the multivariable fixed-effects model to assess incremental fit from the random intercept.

A decision tree classification model was constructed to investigate the predictive capacity of radiologic features for HCC diagnosis. The algorithm recursively partitioned the dataset on the basis of the feature that yielded the highest information gain, maximizing the separation of benign and malignant nodules. To prevent overfitting and enhance interpretability, the tree was limited to a maximum depth of 3 splits. The model is not intended to serve as a predictive tool, but rather as an exploratory analysis to understand the relationships among radiologic features.

## RESULTS

The study included 19 patients with cirrhosis (13 men, 6 women), with a median age of 64 y (interquartile range [IQR], 56–72 y) and a total of 54 LR-3 observations (13 malignant and 41 benign). Each patient had a median of 3 observations (IQR, 2–4 observations). The contrast-enhanced MRI that identified the LR-3 observations was performed a median of 45 d (IQR, 18–84 d) before [^68^Ga]Ga-PSMA-11 PET/MRI. Three patients (15.7%) had concurrent HCC at the time of imaging. Multiple LR-3 observations were present in 14 patients (74%), with a median of 3 observations per patient. The median diameter of all LR-3 observations was 10 mm (IQR, 7–13 mm). Malignant observations had a median diameter of 12 mm (IQR, 9–15 mm), whereas benign observations had a median size of 9.5 mm (IQR, 7–12 mm). All LR-3 observations identified on the index MRI were also visible on the [^68^Ga]Ga-PSMA-11 PET/MRI scan. Treatments details are reported in Table 1.

**TABLE 1.** Locoregional and Surgical Treatments Before and After [^68^Ga]Ga-PSMA-11 PET/MRI

Treatment Timing	*n*	Details
No treatment within 12 mo before [^68^Ga]Ga-PSMA-11 PET/MRI	19	Per study inclusion criteria
Remote locoregional treatment (>12 mo before [^68^Ga]Ga-PSMA-11 PET/MRI)	9	Microwave ablation (*n* = 6), microwave ablation plus SIRT (*n* = 2), SIRT only (*n* = 1)
No prior treatment (>12 mo or within 12 mo of [^68^Ga]Ga-PSMA-11 PET/MRI)	10	—
Treatment after [^68^Ga]Ga-PSMA-11 PET/MRI	3	Liver transplantation (*n* = 1), partial hepatectomy, (*n* = 2; 1 within 2 mo, 1 within 4 mo)
Other treatments during follow-up (12 mo)	0	—

All patients had clinically established cirrhosis. Etiologies included hepatitis B (*n* = 1), cryptogenic cirrhosis (*n* = 1), metabolic dysfunction–associated steatohepatitis (*n* = 1), hepatitis C with metabolic dysfunction–associated steatohepatitis and alcohol consumption (*n* = 1), hepatitis C with alcohol consumption (*n* = 1), hepatitis C alone (*n* = 3), alcohol-related liver disease alone (*n* = 5), and mixed metabolic and alcohol-related liver disease (*n* = 6). Alcohol consumption contributed to cirrhosis in 68% of patients.

Histopathology was used as the reference standard for 21 observations and was obtained a median of 36 d (IQR, 21–72 d) after PET/MRI, whereas follow-up MRI served as the reference standard for 33 observations and was obtained a median of 320 d (IQR, 300–345 d) after PET/MRI. Pathology consisted of percutaneous biopsy (15 observations in 9 patients), partial hepatectomy (3 observations in 1 patient and 1 in another), and liver transplantation specimens (2 observations in 1 patient). Overall, 13 (24%) of 54 LR-3 observations were proven to be HCC (6 by pathology, 7 by imaging follow-up), whereas 41 observations (76%) were benign (15 by pathology, 26 by follow-up imaging). Table 2 summarizes the imaging features and ancillary findings. No observation demonstrated an enhancing capsule.

**TABLE 2.** Mixed-Effects Logistic Regression Results for Imaging Predictors

Variable	β	SE	*z*	*P*	OR	95% CI
Intercept	−2.351	1.176	−1.998	0.046	0.095	−4.656 to −0.045
^68^Ga-PSMA uptake	3.034	1.259	2.411	0.016	4.9	0.567 to 5.502
Arterial phase enhancement	1.463	1.244	1.176	0.239	4.319	−0.975 to 3.900
Portal venous phase washout	0.148	1.207	0.123	0.902	1.159	−2.217 to 2.513
Hepatobiliary phase hypointensity	−0.087	0.983	−0.089	0.929	0.916	−2.013 to 1.839
Diffusion restriction	−0.054	0.996	−0.054	0.957	0.947	−2.006 to 1.898
Observation size	−0.052	0.120	−0.435	0.664	0.949	−0.289 to 0.184

OR = odds ratio.

**FIGURE 1.**
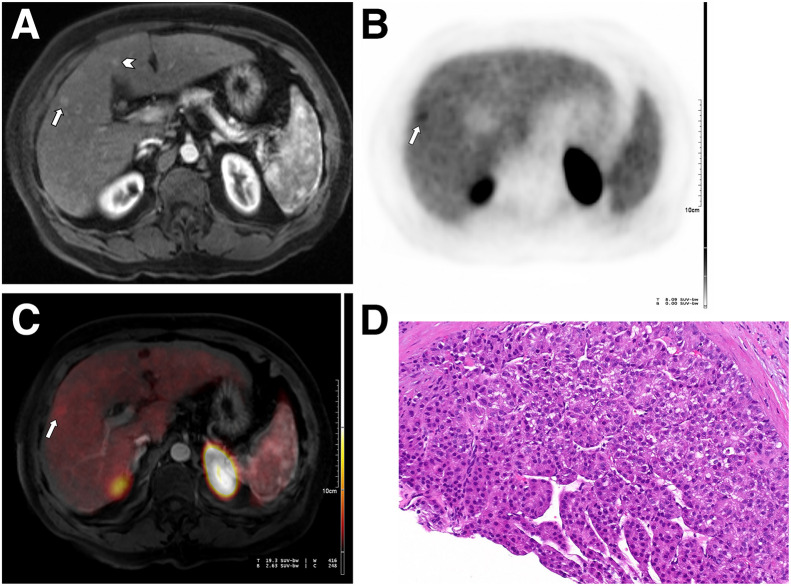
Correlative imaging of 2 LR-3 observations: (A) axial contrast-enhanced arterial phase MRI, (B) axial PET, (C) axial fused arterial phase [^68^Ga]Ga-PSMA-11 PET/MRI, and (D) corresponding pathology. A 7-mm observation in hepatic segment V/VIII (arrow) and 6-mm observation in hepatic segment intravenous (arrowhead) exhibit sharp margins, ovoid shape, and arterial enhancement. Observation in segment V/VIII also demonstrates focal ^68^Ga-PSMA uptake exceeding background liver. Segment V/VIII observation, which was biopsied, revealed hepatocellular carcinoma characterized by thickened trabeculae and endothelial wrapping (H&E, 200X). Observation in segment IV resolved at follow-up imaging.

**FIGURE 2.**
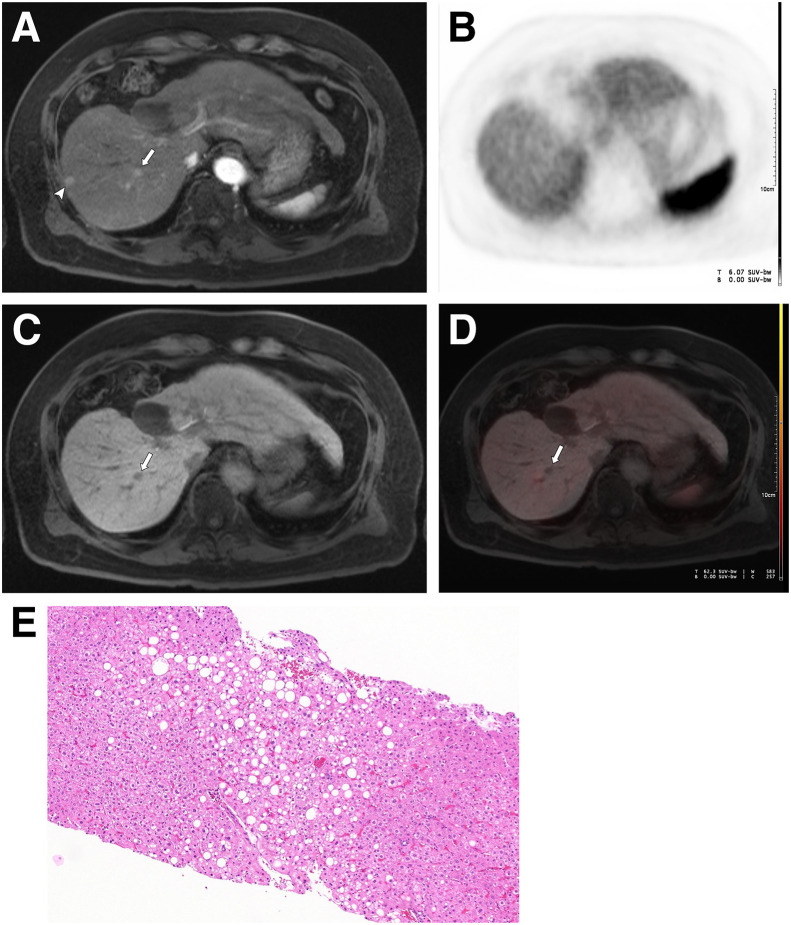
Correlative imaging of 2 LR-3 observations: (A) axial contrast-enhanced arterial phase MRI, (B) axial PET, (C) axial hepatobiliary phase MRI, (D) axial fused hepatobiliary phase [^68^Ga]Ga-PSMA-11 PET/MRI, and (E) corresponding pathology. Sharply marginated, ovoid observations are located in subcapsular segment VIII (arrowhead, 9 mm) and central segment VIII (arrow, 8 mm), both showing arterial phase enhancement. Central observation does not retain hepatobiliary contrast in hepatobiliary phase. Neither observation exhibits ^68^Ga-PSMA uptake. Pathology of central observation showed mild steatosis without significant fibrosis or evidence of HCC (hematoxylin and eosin stain, ×200).

**FIGURE 3.**
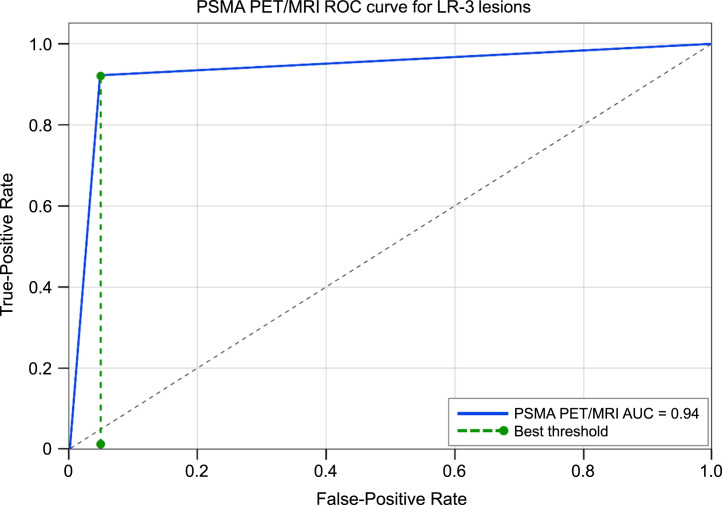
ROC curve demonstrates excellent diagnostic accuracy of [^68^Ga]Ga-PSMA-11 PET/MRI for differentiating malignant from benign LR-3 observations, with area under curve (AUC) of 0.94.

[^68^Ga]Ga-PSMA-11 PET/MRI correctly detected 12 of 13 malignant observations (Fig. 1), corresponding to a sensitivity of 92% (95% CI, 66.7–99.6). The only false-negative observation showed no ^68^Ga-PSMA uptake (supplemental materials, available at http://jnm.snmjournals.org) and was diagnosed as moderately/well-differentiated HCC on pathology. Among benign observations, 39 of 41 were correctly classified (Fig. 2), yielding a specificity of 95% (95% CI, 81.9–99.3). Two observations were false positives (supplemental materials), showing focal uptake; histology demonstrated regenerative nodules with bile duct proliferation, increased vascularity, mild chronic inflammation, and no neoplasm. The overall diagnostic accuracy was 94% (95% CI, 83.1–98.7). The positive predictive value was 86% (95% CI, 60.1–97.5), and negative predictive value was 97% (95% CI, 85.1–99.9). ROC analysis yielded an area under the curve of 0.94 (Fig. 3).

In the stand-alone MRI LI-RADS analysis, which assessed morphologic criteria only, performance was substantially lower: overall accuracy was 54% (95% CI, 40.6–66.3), sensitivity was 69% (95% CI, 42.4–87.3), specificity was 49% (95% CI, 34.3–63.5), positive predictive value was 30% (95% CI, 16.7–47.9), and negative predictive value was 83% (95% CI, 64.1–93.3). McNemar test confirmed a statistically significant improvement with [^68^Ga]Ga-PSMA-11 PET/MRI compared with MRI alone (*P* < 0.001).

In a secondary analysis that allowed observations to be classified as HCC without focal ^68^Ga-PSMA uptake if LI-RADS LR-4 or LR-5 criteria were met, the single false-negative case from the primary analysis was reclassified as malignant because of the presence of non–rim arterial phase hyperenhancement and hepatobiliary phase hypointensity, consistent with LR-4.

**FIGURE 4.**
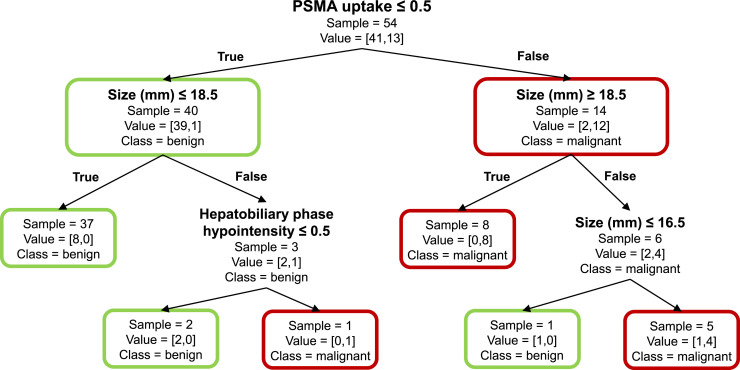
Decision tree model for HCC prediction. Each node within tree provides information about total sample size (“sample”) and class distribution (“value” = [number of benign, number of malignant]). Dominant class in each node is visually indicated by color coding (red for malignant, green for benign).

In multivariable logistic regression, ^68^Ga-PSMA uptake remained the only variable independently associated with malignancy (adjusted odds ratio, 4.9; 95% CI, 1.2–20.0; *P* = 0.028). No other imaging feature reached statistical significance. The mixed-effects logistic regression model, accounting for clustering of observations within patients, confirmed the significance of ^68^Ga-PSMA uptake (β = 3.0; SE = 1.26; *z* = 2.41; *P* = 0.016). A conditional likelihood ratio test demonstrated that inclusion of a random patient intercept significantly improved model fit (likelihood ratio, 5.24; *P* = 0.022). Decision tree analysis identified ^68^Ga-PSMA uptake, observation size, and hepatobiliary phase hypointensity as the main determinants of observation classification, with ^68^Ga-PSMA uptake emerging as the most powerful discriminator (Fig. 4).

## DISCUSSION

In this study, we demonstrated that [^68^Ga]Ga-PSMA-11 PET/MRI shows high diagnostic performance in differentiating malignant from benign LR-3 observations in patients with cirrhosis. LR-3 observations represent an intermediate probability (10%–30%) of malignancy and pose a major clinical challenge, as they frequently require repeated follow-up imaging or biopsy, contributing to patient anxiety, health care costs, and delays in treatment (28).

Reported rates of progression from LR-3 to HCC vary significantly, from 15% to 68.9%, depending on the population, follow-up interval, and imaging modality. Furthermore, imaging alone cannot reliably predict which observations will progress to HCC (5, 27). Advanced MRI features, such as the coexistence of arterial phase hyperenhancement and hepatobiliary phase hypointensity, improve sensitivity but often reduce specificity, emphasizing the need for more accurate biomarkers (29–31).

[^68^Ga]Ga-PSMA-11 PET/MRI demonstrated statistically superior diagnostic performance over stand-alone MRI LI-RADS analysis, supporting its potential as a reliable diagnostic tool. In our study design, observation classification relied on the concordant interpretation of PET and MRI data, integrating metabolic and morphologic information. PET positivity was required to confirm malignancy, as using MRI criteria alone in PET-negative observations could have led to the introduction of false positives because of the intrinsic limitations of LI-RADS categories. These results align with previous studies highlighting the utility of PSMA as a biomarker for HCC (10,18). By providing a more accurate assessment of indeterminate liver observations, [^68^Ga]Ga-PSMA-11 PET/MRI has the potential to influence clinical management, particularly in cases where the true nature of LR-3 observations may impact treatment decisions.

Although the liver typically demonstrates homogeneous and moderate [^68^Ga]Ga-PSMA-11 uptake, focal areas of increased uptake may also occur as a result of benign entities, such as hemangiomas or inflammation, potentially mimicking malignancy. This represents a clinically relevant challenge; approximately 5% of PSMA-positive hepatic observations have been shown to correspond to benign findings rather than HCC or metastases (32,33). Therefore, although [^68^Ga]Ga-PSMA-11 PET/MRI was crucial for identifying HCC within indeterminate LR-3 observations, the additional anatomic and functional information provided by MRI was not negligible. The combination of metabolic and morphologic data in simultaneous PET/MRI helps rule out false-positive results and enhances diagnostic confidence, supporting the use of integrated PET/MRI systems in this clinical setting.

The ROC curve analysis demonstrated a high discriminatory performance of [^68^Ga]Ga-PSMA-11 PET/MRI in differentiating malignant from benign LR-3 observations, with an area under the curve of 0.94. This indicates excellent diagnostic accuracy, with strong differentiation between true positives and true negatives. The steep initial rise in the ROC curve suggests that [^68^Ga]Ga-PSMA-11 PET/MRI has a high true-positive rate with relatively few false positives, aligning with the observed sensitivity of 92% and specificity of 95%. [^68^Ga]Ga-PSMA-11 PET/MRI can reduce diagnostic uncertainty in indeterminate liver observations, potentially allowing for earlier HCC detection and fewer unnecessary follow-up tests or biopsies. These findings align with literature showing PSMA-targeted PET being highly effective for detecting HCC, with a reported sensitivity of 64%–97% and 97% overall accuracy using uptake exceeding background liver activity as the criterion, potentially detecting all HCCs and changing clinical management in 47.5% of cases (17,18).

In this study, PET/MRI was selected not to imply superiority over PET/CT but to explore whether combining PSMA-PET findings with high-resolution liver MRI, within a single examination, could improve detection and characterization of LR-3 observations by reducing false positives and enhancing diagnostic confidence. Our observed false negative was a moderately/well-differentiated HCC that lacked PSMA accumulation. This aligns with some literature suggesting lower PSMA expression in well-differentiated HCC, although the overall correlation is debated (15,19,20). Although MRI features would have classified the lesion as LR-4 (probable HCC), the study’s overall results showed that focal PSMA uptake was superior for detecting HCC compared with reliance solely on LI-RADS criteria.

Our 2 false-positive cases, which showed irregular, indistinct [^68^Ga]Ga-PSMA-11 uptake (unlike the nodular true positives), were located near prior SIRT (37 mo) and SIRT plus ablation (15 mo). Pathology revealed regenerative nodules and inflammatory changes, highlighting a limitation in patients after local therapy, especially SIRT. These findings, coupled with the variable and conflicting literature on the duration of SIRT-induced liver injury and inflammation and the onset of sinusoidal obstruction syndrome, indicate that [^68^Ga]Ga-PSMA-11 PET/MRI requires careful interpretation in this patient subset (34–37).

### Limitations

Study limitations included the single-institution design and small cohort, which restricted multivariate analysis. We addressed this by using a mixed-effects model to account for nodule clustering and reduce potential bias. We did not report interreader agreement, as we used a 3-person consensus method, a common practice used to establish robust diagnostic potential. Although this consensus approach may yield higher accuracy than a single-reader approach, it provides a valuable benchmark for future research, training, and guideline development. Moreover, although the image analyses were performed at least 4 wk apart and in random order to reduce recall bias, this interval may not have eliminated it.

For 21 observations, pathology served as the reference standard. To minimize potential sampling issues, percutaneous biopsies were carefully guided by a combination of contrast-enhanced ultrasound/CT and aligned with MRI images and anatomic landmarks. This meticulous approach ensured accurate sampling of the target observations. During pathology analysis, equal care was taken to specifically identify and analyze the corresponding MRI observations on the surgical specimen.

Despite the limited number of patients, this study revealed a potential increase in diagnostic accuracy in an area of uncertainty, where conventional MRI or contrast-enhanced ultrasound failed to reach a diagnostic accuracy rate of greater than 80%. As this was a preliminary study, we did not assess interreader reliability and instead used consensus reads.

Another limitation is the inherent biologic complexity of HCC. Because the study captured observations at a single point in time, it may not reflect the potential progression of benign lesions into HCC. Additionally, the patient population originated from a tertiary referral center, potentially limiting generalizability, although the observed malignancy rate of LR-3 observations (24%) is consistent with that found in prior literature. Future multicenter studies should validate these findings and assess the cost-effectiveness of [^68^Ga]Ga-PSMA-11 PET/MRI in routine practice, which could potentially reduce follow-up imaging and biopsies.

## CONCLUSION

[^68^Ga]Ga-PSMA-11 PET/MRI demonstrated high accuracy in characterizing LR-3 observations in patients with cirrhosis, outperforming stand-alone MRI LI-RADS criteria. Its high negative predictive value suggests that it may help reduce unnecessary follow-up imaging and biopsies, minimizing patient burden and health care costs. Although false-positive results may occur in previously treated liver segments, integration of metabolic ^68^Ga-PSMA uptake with high-resolution MRI improves diagnostic confidence. These preliminary findings warrant validation in larger, multicenter cohorts and may support future incorporation of [^68^Ga]Ga-PSMA-11 PET/MRI into diagnostic algorithms for indeterminate liver observations.

## DISCLOSURE

The in-kind donation of ^68^Ga-gozetotide (Illuccix) vials provided by Telix Pharmaceuticals contributed to the conduct of this study. However, Telix had no role in the conception, design, data collection, analysis, interpretation, or writing of the manuscript. Furthermore, Telix had no involvement in the decision to submit this article for publication. The contribution of Telix was limited to the provision of Illuccix vials, and it did not participate in any other aspect of the study or manuscript preparation. No other potential conflict of interest relevant to this article was reported.

## Notes

KEY POINTS **QUESTION:** Can [^68^Ga]Ga-PSMA-11 PET/MRI accurately differentiate between benign and malignant LI-RADS 3 liver observations in patients with cirrhosis? **PERTINENT FINDINGS:** In this prospective study of 19 patients with cirrhosis with 54 LR-3 observations, [^68^Ga]Ga-PSMA-11 PET/MRI demonstrated a sensitivity of 92%, specificity of 95%, and overall diagnostic accuracy of 94% in identifying HCC. [^68^Ga]Ga-PSMA-11 uptake was the only independent imaging predictor of malignancy. **IMPLICATIONS FOR PATIENT CARE:** [^68^Ga]Ga-PSMA-11 PET/MRI may improve diagnostic confidence in indeterminate liver observations, potentially reducing the need for invasive biopsy and unnecessary follow-up in patients with cirrhosis.

## References

[1] Tan EY Danpanichkul P Yong JN. Liver cancer in 2021: global burden of disease study. J Hepatol. 2024;82:851–860. 39481652 10.1016/j.jhep.2024.10.031

[2] Chernyak V Fowler KJ Kamaya A. Liver Imaging Reporting and Data System (LI-RADS) version 2018: imaging of hepatocellular carcinoma in at-risk patients. Radiology. 2018;289:816–830. 30251931 10.1148/radiol.2018181494 PMC6677371

[3] Lee S Kim Y-Y Shin J. Percentages of hepatocellular carcinoma in LI-RADS categories with CT and MRI: a systematic review and meta-analysis. Radiology. 2023;307:e220646. 36625748 10.1148/radiol.220646

[4] Dunn C Lin B Rich NE Patel MS Gopal P Singal AG. Correlation of LI-RADS 3 or 4 observations with histopathologic diagnosis in patients with cirrhosis. Clin Gastroenterol Hepatol. 2023;21:1351–1353.e2. 35307596 10.1016/j.cgh.2022.03.009 PMC9481748

[5] Kanneganti M Marrero JA Parikh ND. Clinical outcomes of patients with Liver Imaging Reporting and Data System 3 or Liver Imaging Reporting and Data System 4 observations in patients with cirrhosis: a systematic review. Liver Transpl. 2022;28:1865–1875. 35980600 10.1002/lt.26562 PMC9669163

[6] Marrero JA Kulik LM Sirlin CB. Diagnosis, staging, and management of hepatocellular carcinoma: 2018 practice guidance by the American Association for the Study of Liver Diseases. Hepatology. 2018;68:723–750. 29624699 10.1002/hep.29913

[7] Tang A Bashir MR Corwin MT.; LI-RADS Evidence Working Group. Evidence supporting LI-RADS major features for CT- and MR imaging–based diagnosis of hepatocellular carcinoma: a systematic review. Radiology. 2018;286:29–48. 29166245 10.1148/radiol.2017170554 PMC6677284

[8] Siegel RL Miller KD Wagle NS Jemal A. Cancer statistics, 2023. CA Cancer J Clin. 2023;73:17–48. 36633525 10.3322/caac.21763

[9] Frenette C Mendiratta-Lala M Salgia R Wong RJ Sauer BG Pillai A. ACG clinical guideline: focal liver lesions. Am J Gastroenterol. 2024;119:1235–1271. 38958301 10.14309/ajg.0000000000002857

[10] Eiber M Fendler WP Rowe SP. Prostate-specific membrane antigen ligands for imaging and therapy. J Nucl Med. 2017;58:67S–76S. 28864615 10.2967/jnumed.116.186767

[11] Schmidt LH Heitkötter B Schulze AB. Prostate specific membrane antigen (PSMA) expression in non-small cell lung cancer. PLoS One. 2017;12:e0186280. 29077706 10.1371/journal.pone.0186280 PMC5659610

[12] Bychkov A Vutrapongwatana U Tepmongkol S Keelawat S. PSMA expression by microvasculature of thyroid tumors—potential implications for PSMA theranostics. Sci Rep. 2017;7:5202. 28701709 10.1038/s41598-017-05481-z PMC5507885

[13] Heitkötter B Trautmann M Grünewald I. Expression of PSMA in tumor neovasculature of high grade sarcomas including synovial sarcoma, rhabdomyosarcoma, undifferentiated sarcoma and MPNST. Oncotarget. 2017;8:4268–4276. 28002805 10.18632/oncotarget.13994 PMC5354830

[14] Jiao J Jiao D Yang F. Galectin-9 expression predicts poor prognosis in hepatitis B virus-associated hepatocellular carcinoma. Aging (Albany NY). 2022;14:1879–1890. 35202002 10.18632/aging.203909 PMC8908941

[15] Tolkach Y Goltz D Kremer A. Prostate-specific membrane antigen expression in hepatocellular carcinoma: potential use for prognosis and diagnostic imaging. Oncotarget. 2019;10:4149–4160. 31289613 10.18632/oncotarget.27024 PMC6609242

[16] Kmeid M Park YN Chung T. PSMA immunohistochemistry in hepatic neoplasms. Am J Surg Pathol. 2022;46:1688–1699. 36190927 10.1097/PAS.0000000000001971

[17] Kesler M Levine C Hershkovitz D. ^68^Ga-labeled prostate-specific membrane antigen is a novel PET/CT tracer for imaging of hepatocellular carcinoma: a prospective pilot study. J Nucl Med. 2019;60:185–191. 30002112 10.2967/jnumed.118.214833

[18] Hirmas N Leyh C Sraieb M. ^68^ Ga-PSMA-11 PET/CT improves tumor detection and impacts management in patients with hepatocellular carcinoma. J Nucl Med. 2021;62:1235–1241. 33509970 10.2967/jnumed.120.257915 PMC8882890

[19] Thompson SM Suman G Torbenson MS. PSMA as a theranostic target in hepatocellular carcinoma: immunohistochemistry and ^68^Ga‐PSMA‐11 PET using cyclotron‐produced ^68^Ga. Hepatol Commun. 2022;6:1172–1185. 34783177 10.1002/hep4.1861 PMC9035563

[20] Qin C Song X Sun S. [^68^Ga]Ga-PSMA-617 PET/MRI for imaging patients suspected of hepatocellular carcinoma. Eur J Nucl Med Mol Imaging. 2025;52:1278–1290. 39570398 10.1007/s00259-024-06973-7

[21] Pan J Li W Gu L Liu C Zhang K Hong G. Performance of adding hepatobiliary phase image in magnetic resonance imaging for detection of hepatocellular carcinoma: a meta-analysis. Eur Radiol. 2022;32:7883–7895. 35579711 10.1007/s00330-022-08826-z

[22] Kim Y-Y Lee S Shin J. Diagnostic performance of CT versus MRI Liver Imaging Reporting and Data System category 5 for hepatocellular carcinoma: a systematic review and meta-analysis of comparative studies. Eur Radiol. 2022;32:6723–6729. 35849177 10.1007/s00330-022-08985-z

[23] Chen X Li M Guo R. The diagnostic performance of contrast-enhanced CT versus extracellular contrast agent-enhanced MRI in detecting hepatocellular carcinoma: direct comparison and a meta-analysis. Abdom Radiol (NY). 2022;47:2057–2070. 35312822 10.1007/s00261-022-03484-7

[24] Cococcia S Dutta P Moghim M. The fate of indeterminate liver lesions: what proportion are precursors of hepatocellular carcinoma? BMC Gastroenterol. 2022;22:118. 35272611 10.1186/s12876-022-02135-x PMC8908619

[25] Niu Z-S Niu X-J Wang W-H Zhao J. Latest developments in precancerous lesions of hepatocellular carcinoma. World J Gastroenterol. 2016;22:3305–3314. 27022212 10.3748/wjg.v22.i12.3305 PMC4806188

[26] Park YN. Update on precursor and early lesions of hepatocellular carcinomas. Arch Pathol Lab Med. 2011;135:704–715. 21631263 10.5858/2010-0524-RA.1

[27] Darnell A Rimola J Belmonte E. Evaluation of LI-RADS 3 category by magnetic resonance in US-detected nodules ≤ 2 cm in cirrhotic patients. Eur Radiol. 2021;31:4794–4803. 33409789 10.1007/s00330-020-07457-6

[28] Lee S Kim Y-Y Shin J. CT and MRI Liver Imaging Reporting and Data System version 2018 for hepatocellular carcinoma: a systematic review with meta-analysis. J Am Coll Radiol. 2020;17:1199–1206. 32640250 10.1016/j.jacr.2020.06.005

[29] Jeon SK Joo I Bae JS Park S-J Lee JM. LI-RADS v2018: how to appropriately use ancillary features in category adjustment from intermediate probability of malignancy (LR-3) to probably HCC (LR-4) on gadoxetic acid–enhanced MRI. Eur Radiol. 2022;32:46–55. 34132875 10.1007/s00330-021-08116-0

[30] Siu Xiao T Kuon Yeng Escalante CM Tahmasebi A. Combining CEUS and CT/MRI LI-RADS major imaging features: diagnostic accuracy for classification of indeterminate liver observations in patients at risk for HCC. Abdom Radiol (NY). October 2024;50:2066–2077. 39438285 10.1007/s00261-024-04625-w PMC11991985

[31] Lin B Zhang W Jiang Y. Diagnostic performance of LR-5 based on hypointensity on Gd-EOB-DTPA–enhanced MRI in the hepatobiliary phase for sHCC using LI-RADS v2018 criteria. Clin Radiol. 2025;81:106784. 39799836 10.1016/j.crad.2024.106784

[32] Ladrón-de-Guevara D Canelo A Piottante A Regonesi C. False-positive ^18^F–prostate-specific membrane antigen–1007 PET/CT caused by hepatic multifocal inflammatory foci. Clin Nucl Med. 2021;46:e80–e83. 33234935 10.1097/RLU.0000000000003425

[33] Bhardwaj H Stephens M Bhatt M Thomas PA. Prostate-specific membrane antigen PET/CT findings for hepatic hemangioma. Clin Nucl Med. 2016;41:968–969. 27749413 10.1097/RLU.0000000000001384

[34] Ricke J Schinner R Seidensticker M. Liver function after combined selective internal radiation therapy or sorafenib monotherapy in advanced hepatocellular carcinoma. J Hepatol. 2021;75:1387–1396. 34454995 10.1016/j.jhep.2021.07.037

[35] Alnammi M Wortman J Therrien J Afnan J. MRI features of treated hepatocellular carcinoma following locoregional therapy: a pictorial review. Abdom Radiol (NY). 2022;47:2299–2313. 35524803 10.1007/s00261-022-03526-0

[36] Miller FH Lopes Vendrami C Gabr A. Evolution of radioembolization in treatment of hepatocellular carcinoma: a pictorial review. Radiographics. 2021;41:1802–1818. 34559587 10.1148/rg.2021210014

[37] Justinger C Gruden J Kouladouros K. Histopathological changes resulting from selective internal radiotherapy (SIRT). J Surg Oncol. 2018;117:1084–1091. 29448307 10.1002/jso.24967

